# Geographic and temporal variation in racial and ethnic disparities in SARS-CoV-2 positivity between February 2020 and August 2021 in the United States

**DOI:** 10.1038/s41598-021-03967-5

**Published:** 2022-01-07

**Authors:** Jacqueline M. Ferguson, Amy C. Justice, Thomas F. Osborne, Hoda S. Abdel Magid, Amanda L. Purnell, Christopher T. Rentsch

**Affiliations:** 1 Center for Innovation to Implementation, VA Palo Alto Healthcare System, US Department of Veterans Affairs, MDP-152, 795 Willow Rd, Menlo Park, CA 94025 USA; 2 Stanford Center for Population Health Sciences, Stanford University School of Medicine, Stanford, CA USA; 3 VA Connecticut Healthcare System, US Department of Veterans Affairs, West Haven, CT USA; 4 School of Public Health, Yale, New Haven, CT USA; 5 Department of Internal Medicine, Yale School of Medicine, New Haven, CT USA; 6 Department of Radiology, Stanford University School of Medicine, Stanford, CA USA; 7 Department of Epidemiology and Population Health, Stanford University, Stanford, CA USA; 8 Public Health Program, Santa Clara University, Santa Clara, CA USA; 9 VA Central Office, US Department of Veterans Affairs, Washington, DC USA; 10 Faculty of Epidemiology and Population Health, London School of Hygiene and Tropical Medicine, London, UK

**Keywords:** Viral infection, Epidemiology, Epidemiology, Risk factors

## Abstract

The coronavirus pandemic has disproportionally impacted racial and ethnic minority communities in the United States. Patterns of these disparities may be changing over time as outbreaks occur in different communities. Utilizing electronic health record data from the US Department of Veterans Affairs (VA), we estimated odds ratios, stratified by time period and region, for testing positive among 1,313,402 individuals tested for SARS-CoV-2 between February 12, 2020 and August 16, 2021 at VA medical facilities. We adjusted for personal characteristics (sex, age, rural/urban residence, VA facility) and a wide range of clinical characteristics that have been evaluated in prior SARS-CoV-2 reports and could potentially explain racial/ethnic disparities in SARS-CoV-2. Our study found racial and ethnic disparities for testing positive were most pronounced at the beginning of the pandemic and decreased over time. A key finding was that the disparity among Hispanic individuals attenuated but remained elevated, while disparities among Asian individuals reversed by March 1, 2021. The variation in racial and ethnic disparities in SARS-CoV-2 positivity by time and region, independent of underlying health status and other demographic characteristics in a nationwide cohort, provides important insight for strategies to prevent further outbreaks.

## Introduction

The coronavirus pandemic has disproportionally impacted racial and ethnic minority communities in the United States^1–3^. Evidence has highlighted the vast disparities in SARS-CoV-2 infection and subsequent COVID-19 among persons who were Black, Hispanic or Latino, Native Hawaiian/Pacific Islander, American Indian/Alaska Native, or Asian^4–12^. Recently, additional analyses have suggested that racial and ethnic disparities may be changing over time as outbreaks spread from racially and ethnically diverse metropolitan centers to more rural and less diverse areas^4,5,13,14^. In this report, we updated our previous analyses^4,5^ to evaluate changes in disparities for testing positive with SARS-CoV-2 over the first 18 months of the pandemic and by geographic region in the largest integrated healthcare system in the United States.

## Methods

Utilizing national electronic health record data from the US Department of Veterans Affairs (VA), we conducted a retrospective cohort analysis of all Veterans tested for SARS-CoV-2 in VA medical facilities between February 12, 2020 and August 16, 2021. Methods have been previously described in detail^4,5^. In brief, SARS-CoV-2 tests were identified using a text searching algorithm of laboratory results at VA for terms consistent with SARS-CoV-2 or COVID-19. For individuals with multiple tests, we selected the first positive test. For those without a positive test during the study period, we selected the first negative test. Analysis of test samples was performed in VA, state public health, and commercial laboratories using FDA Emergency Use Authorization-approved SARS-CoV-2 assays. Nearly all the tests utilized nasopharyngeal swabs. We did not include antibody tests in this analysis.

We calculated the crude prevalence of testing positive for SARS-CoV-2 by time and race/ethnicity and calculated confidence intervals using the normal approximation. We used multivariable logistic regression to estimate odds ratios (OR) and 95% confidence intervals (CI) for testing positive for SARS-CoV-2 for Black, Hispanic or Latino (Hispanic), Asian, American Indian/Alaska Native, Native Hawaiian/Pacific Islander, and people of mixed race (Mixed), relative to White individuals. Race and ethnicity were self-reported. Individuals who reported Hispanic ethnicity were included in the Hispanic group regardless of any other self-reported race. A small proportion (4.0%) of individuals tested for SARS-CoV-2 with missing race/ethnicity information were excluded from analysis. All models were adjusted for other personal characteristics (sex, age, rural/urban residence) and a wide range of clinical characteristics that have been evaluated in prior SARS-CoV-2 reports and could potentially explain racial/ethnic disparities in SARS-CoV-2 positivity^4,5,15,16^. Clinical characteristics included baseline comorbidities (asthma, cancer, chronic kidney disease, chronic obstructive pulmonary disease, diabetes mellitus, hypertension, liver disease, vascular disease), substance use (alcohol consumption, alcohol use disorder, smoking status), medication history (angiotensin converting enzyme inhibitor, angiotensin II receptor blocker). Baseline for personal and clinical characteristics was defined as the date of specimen collection for the selected SARS-CoV-2 test. Models were additionally conditioned on VA site of care.

Models were a priori stratified into five waves based on the temporal distribution of SARS-CoV-2 cases nationally: February 12–May 31, 2020 (wave 1); June 1–September 30, 2020 (wave 2); October 1, 2020–February 28, 2021 (wave 3); March 1, 2021–June 30, 2021 (wave 4); and July 1, 2021–August 16, 2021 (wave 5). Due to the large size of the third national wave, we split this period into two waves containing roughly equal numbers of SARS-CoV-2 cases (October 1–December 11, 2020 (wave 3a); and December 12, 2020–February 28, 2021 (wave 3b).

To evaluate regional differences by time, models were further stratified by US Census region (i.e., West, South, Midwest, and Northeast). Odds ratios in strata by time and region with less than five cases were not reported due to potential privacy concerns and instability of statistical estimates. Data analysis was performed using SAS version 9.4 (SAS Institute, Cary, NC).

### Ethics

This study was approved by the institutional review boards of VA Connecticut Healthcare System (VA AJ0013) and Yale University (1506016006). It has been granted a waiver of informed consent and is Health Insurance Portability and Accountability Act compliant.

### Disclaimer

Views expressed are those of the authors and the contents of this article do not represent the views of the US Department of Veterans Affairs or the United States Government.

## Results

Of 1,313,402 individuals tested for SARS-CoV-2 in the VA between February 2020 and August 2021, there were 144,597 (11.0%) who tested positive for SARS-CoV-2 (Table 1). All non-White groups except for Asian individuals had higher crude prevalence of positive tests than White individuals (10.2%), with the largest differences observed among Black (12.2%), Hispanic (14.1%), and American Indian/Alaska Native (12.0.%) groups. By region, the crude prevalence of positive tests was highest in West (12.3%) and lowest in South (9.6%).

**Table 1:** Characteristics of all individuals tested and tested positive for SARS-CoV-2 between February 12, 2020 and August 16, 2021 in the US Department of Veterans Affairs (VA).

	No. tested	No. tested positive	(row%)
Sample size	1,313,402	144,597	(11.0)
**Race/ethnicity**			
White	846,678	86,164	(10.2)
Black	304,893	37,159	(12.2)
Hispanic	115,012	16,219	(14.1)
Asian	15,422	1519	(9.8)
American Indian/Alaska native	9120	1096	(12.0)
Native Hawaiian/Pacific islander	10,008	1125	(11.2)
Mixed	12,269	1315	(10.7)
**Age, years**			
20–39	164,579	23,934	(14.5)
40–49	130,658	18,011	(13.8)
50–59	220,712	27,046	(12.3)
60–69	315,548	30,730	(9.7)
70–79	374,750	34,022	(9.1)
≥ 80	107,155	10,854	(10.1)
**Sex**			
Female	143,523	15,369	(10.7)
Male	1,169,879	129,228	(11.0)
**Region**			
West	262,898	32,441	(12.3)
South	159,668	15,324	(9.6)
Northeast	598,551	67,287	(11.2)
Midwest	292,285	29,545	(10.1)
**Wave**			
1: February 12–May 31, 2020	114,584	7995	(7.0)
2: June 1–September 30, 2020	378,048	23,632	(6.3)
3a: October 1–December 11, 2020	261,912	39,372	(15.0)
3b: December 12, 2020–February 28, 2021	215,861	44,190	(20.5)
4: March 1–June 30, 2021	250,968	14,498	(5.8)
5: July 1–August 16, 2021	92,029	14,910	(16.2)

**Figure 1:**
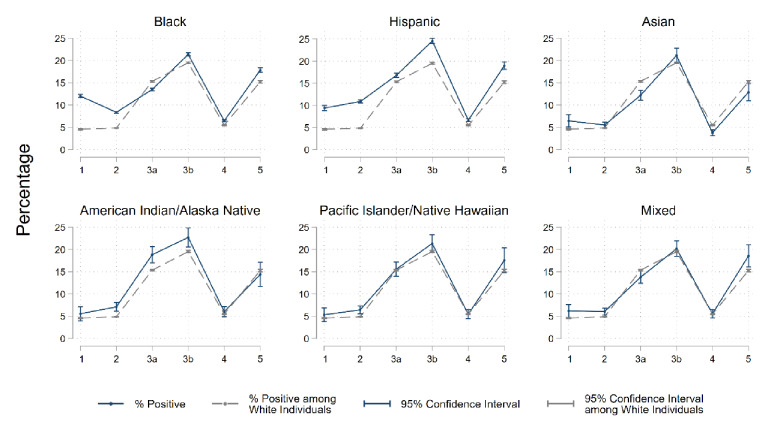
Unadjusted race and ethnicity specific SARS-CoV-2 test positivity percentage by wave between February 12, 2020 and August 16, 2021 in the US Department of Veterans Affairs (VA). Notes: Wave 1 (February 12–May 31, 2020); Wave 2 (June 1–September 30, 2020); Wave 3a (October 1–December 11, 2020); Wave 3b (December 12, 2020–February 28, 2021), Wave 4 (March 1–June 30, 2021; Wave 5 (July 1–August 16, 2021).

Individuals who were younger or male had a slightly higher crude prevalence of positive tests than those who were older or female. Over time, the prevalence of positive tests increased from 7.0% in wave 1, 6.3% in wave 2, 15.0% in wave 3a, and peaked at 20.5% in wave 3b. There was a marked decrease in the crude prevalence for positive tests in wave 4 to 5.8%, followed by a large increase to 16.2% in wave 5. In wave 1, all racial and ethnic minority groups had a higher unadjusted percentage of positive tests relative to White individuals, with the highest percentage observed among Black individuals at 12.0% (Fig. 1). In wave 3b, the highest percentage was observed among Hispanic individuals at 24.5%. By the end of wave 5, all racial and ethnic minority groups had a higher unadjusted percentage of positive tests relative to White individuals (15.2%), with the exception of Asian individuals who had the lowest at 12.9%. Over the study period, White individuals experienced the steepest increase in test positivity percentage (a 3.2-fold relative increase from 4.6% in wave 1 to 19.5% in the peak wave 3b and a 2.3-fold relative increase overall from 4.6% in wave 1 to 15.2% in wave 5) compared to increases observed in all other racial and ethnic minority groups.

**Figure 2:**
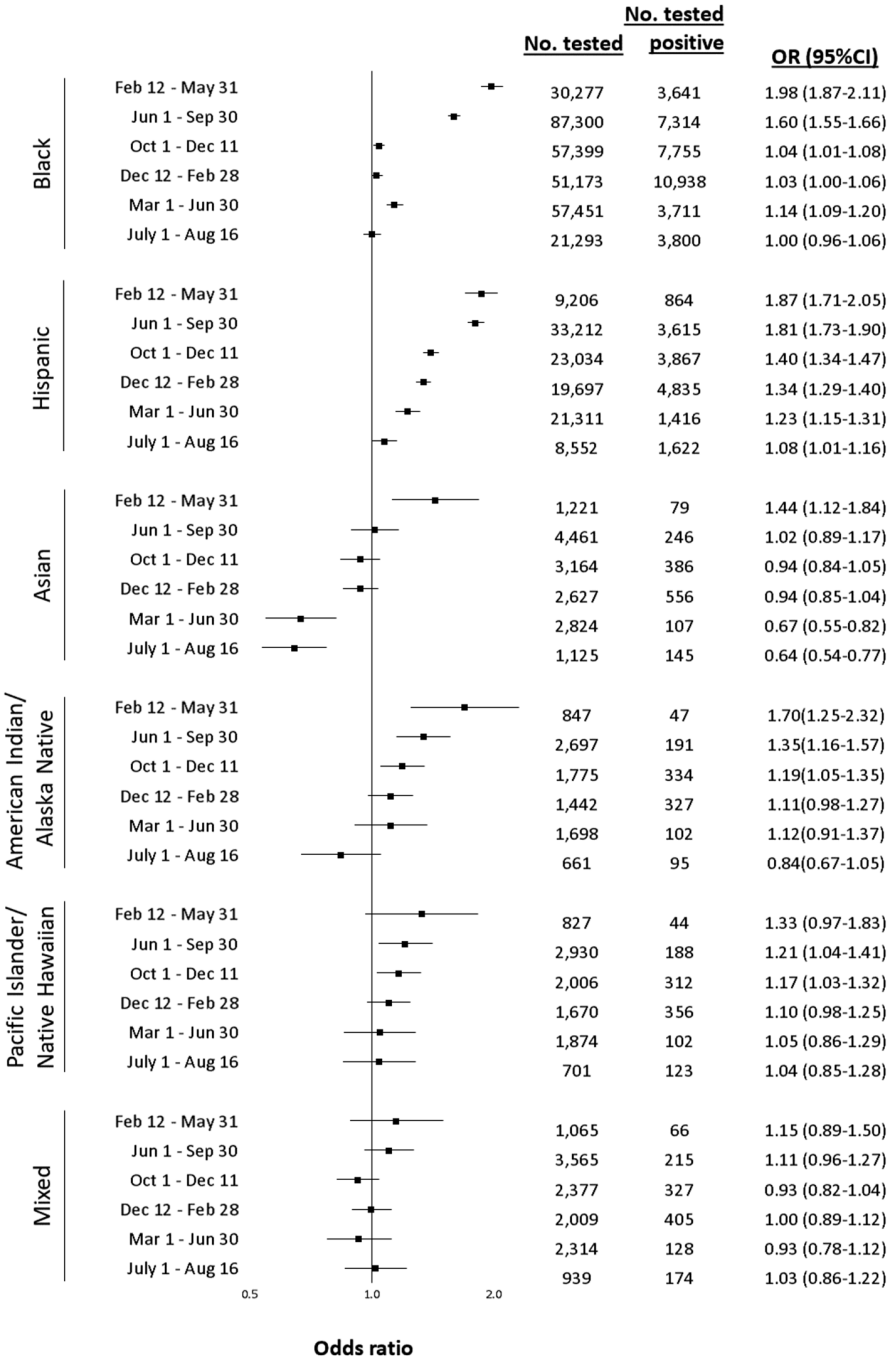
Adjusted racial and ethnic disparities in testing positive for SARS-CoV-2 between February 12, 2020 and August 16, 2021, by wave of the pandemic in the US Department of Veterans Affairs (VA). Notes: Referent group for all comparisons is White. Model conditioned on site of care and adjusted for sex, age, rural/urban residence, and baseline comorbidity (asthma, cancer, chronic kidney disease, chronic pulmonary disease, diabetes mellitus, hypertension, liver disease, vascular disease) substance use (alcohol consumption, alcohol use disorder, smoking status) , and medication history (angiotensin, converting enzyme inhibitor, angiotensin II receptor blocker). *OR* odd ratio, *CI* confidence interval.

After adjustment for personal and clinical characteristics, those who were Black (OR 1.18, 95% CI 1.17–1.20), Hispanic (1.42, 1.39–1.44), American Indian/Alaska Native (1.14, 1.07–1.21), or Native Hawaiian/Pacific Islander (1.13, 1.06–1.20) had elevated odds of testing positive, with no evidence of disparities among people of Mixed race (0.99, 0.94–1.05) compared to White individuals over the entire study period. Asian individuals had lower odds of testing positive compared to White individuals (0.90, 0.86–0.95) over the entire study period. However, there was substantial variation over time. Disparities for testing positive decreased for all racial and ethnic minorities over the study period (Fig. 2) with the largest disparities present in wave 1. In wave 1, disparities in test positivity were observed among Black (1.98, 1.87–2.11), Hispanic (1.87, 1.71–2.05), Asian (1.44, 1.12–1.84), and American Indian/Alaska Native (1.70, 1.25–2.32) individuals compared to White individuals. There was weak evidence of a disparity for testing positive among Native Hawaiian/Pacific Islander individuals (1.33, 0.97–1.83) and no observed disparity among people of mixed race (1.15, 0.89–1.50) in wave 1. By wave 3b, the peak of test positivity, disparities were not observed among any racial or ethnic minority group, apart from Hispanic individuals (1.34, 1.29–1.40). A notable decrease in test positivity disparity was seen among Black individuals, from a near doubling of odds for testing positive in wave 1 (1.98, 1.87–2.11) to only marginally elevated odds by wave 3b (1.03, 1.00–1.06) compared to White individuals. There was a slight elevation in the odds among Black individuals for wave 4, but by wave 5 there was no observed dispairity in testing positive compared to White individuals. While Asian and American Indian/Alaska Native individuals had increased odds of testing positive in wave 1, this association attenuated rapidly and reversed for some. By the end of wave 4, Asian individuals had a 33% lower odds of testing positive (0.67, 0.55–0.82) compared to White individuals, which persisted into wave 5.

**Figure 3:**
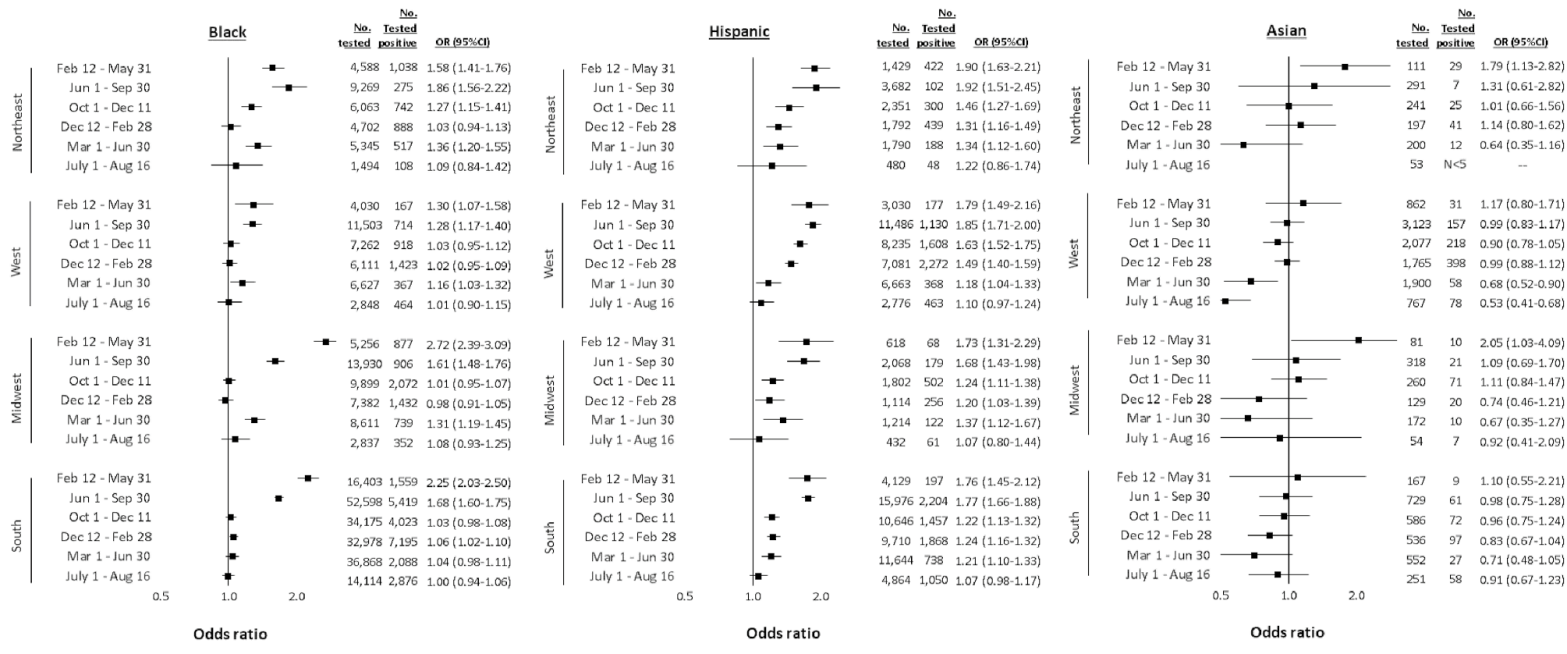
Adjusted racial and ethnic disparities in testing positive for SARS-CoV-2 between February 12, 2020 and August 16, 2021 in the US Department of Veterans Affairs (VA), by US Census region among Black, Hispanic, and Asian individuals. Notes: Referent group for all comparisons is White. Strata with fewer than five positive cases are censored due to potential privacy concerns and instability of statistical estimates. Models conditioned on site of care and adjusted for sex, age, rural/urban residence, and baseline comorbidity (asthma, cancer, chronic kidney disease, chronic obstructive pulmonary disease, diabetes mellitus, hypertension, liver disease, vascular disease), substance use (alcohol consumption, alcohol use disorder, smoking status), and medication history (angiotensin converting enzyme inhibitor, angiotensin II receptor blocker). *OR* odds ratio, *CI* confidence interval.

**Figure 4:**
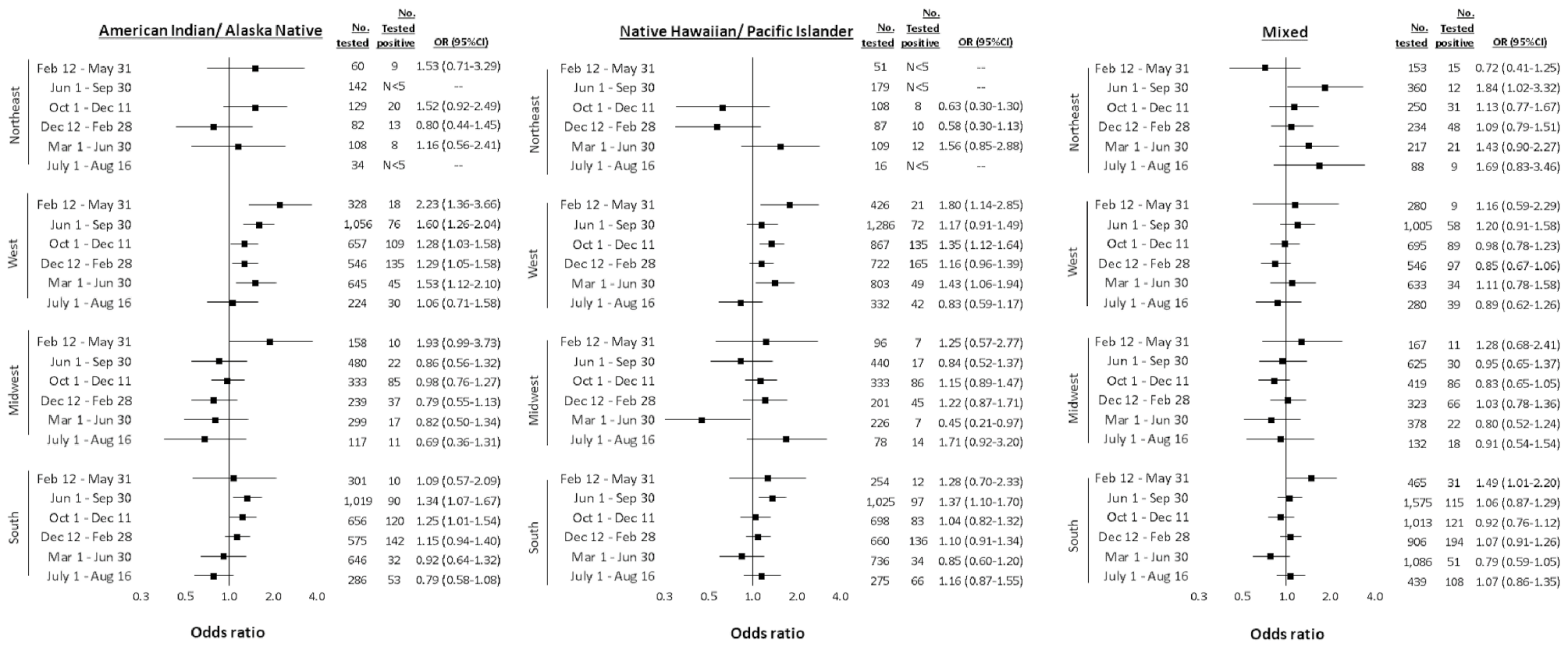
Adjusted racial and ethnic disparities in testing positive for SARS-CoV-2 between February 12, 2020 and August 16, 2021 in the US Department of Veterans Affiars (VA), by US Census region among Mixed, Native Hawaiian/Pacific Islander, and American Indian/Alaska Native individuals. Notes: Referent group for all comparisons is White. Strata with fewer than five positive cases are censored due to potential privacy concerns and instability of statistical estimates. Models conditioned on site of care and adjusted for sex, age, rural/urban residence, and baseline comorbidity (asthma, cancer, chronic kidney disease, chronic obstructive pulmonary disease, diabetes mellitus, hypertension, liver disease, vascular disease), substance use (alcohol consumption, alcohol use disorder, smoking status), and medication history (angiotensin converting enzyme inhibitor, angiotensin II receptor blocker). *OR* odds ratio, *CI* confidence interval.

We found some evidence of regional variation in the disparity for testing positive by time. While wave 1 disparities for Black individuals were found in all regions, they were notably higher in the Midwest (2.72, 2.39–3.09) and the South (2.25, 2.03–2.50) compared with the Northeast (1.58, 1.41–1.76) and the West (1.30, 1.07, 1.58) (Fig. 3). Disparities for testing positive among Hispanic individuals were present in all regions across all time points, apart from wave 5. We found evidence of disparities for Asian individuals in wave 1 in the Northeast (1.79, 1.13–2.82) and Midwest (2.05, 1.03–4.09). American Indian/Alaska Native individuals had elevated odds of testing positive in the West in all time periods apart from wave 5, with the highest odds observed in wave 1 (2.23, 1.36–3.66) (Fig. 4). There were no discernable patterns in regional disparities for Native Hawaiian/Pacific Islander individuals or those of Mixed race; however, these groups had relatively small numbers of events when stratified by time and region.

## Discussion

Our nationwide study utilizing electronic health record data from the VA found that racial and ethnic disparities for testing positive for SARS-CoV-2 were most pronounced at the beginning of the pandemic and that these disparities decreased over time, even after accounting for a wide range of personal and clinical characteristics. By the end of the first 18 months of the pandemic, disparities for testing positive were attenuated but remained elevated for Hispanic individuals and were no longer observed for any other group. This attenuation in disparities appears to be due, at least in part, to a stronger relative increase in the test positivity percentage among White individuals over time rather than a decline in test positivity among racial and ethnic minority groups, which may be driven by the pandemic generalizing from diverse metropolitan areas to less diverse rural areas and, for the later time periods, differential physical distancing practices, and differential vaccination uptake by race and ethnicity^4,5,13,14,17^.

Our findings on disparities for testing positive among Black and Hispanic individuals in the first months of the pandemic have been demonstrated previously^1–5^. This study extended previously published models to evaluate patterns in disparities over the first full year and a half of the pandemic. A novel finding was that disparities for testing positive dramatically attenuated and were no longer observed by August 2021 among all racial and ethnic groups apart from Hispanic individuals. This disparity among Hispanic individuals was observed across all geographic regions between February 2020 and June 2021 and persisted after September 2020 most strongly in the West. A deeper understanding of the mechanism for this association is needed but may be due to the lack of nationwide media coverage and targeted, culturally-responsive, and language-accessible outreach and partnerships with Hispanic communities in the United States during that time. Hispanic individuals are also overrepresented in essential and frontline jobs, which increases their likelihood of SARS-CoV-2 exposure and they may face barriers (e.g., precarious employment or financial limitations) to taking sick leave that would help reduce the spread of SARS-CoV-2^18^.

Another novel finding was the identification of increased odds of testing SARS-CoV-2 positive among Asian individuals in the first wave of the pandemic, which was obscured in the time-pooled model. We also found that Asian individuals had a lower odds of testing positive than White individuals between March and August 2021. One hypothesis for the reduced odds of testing positive among Asian groups is the higher rate of vaccination among Asian individuals compared with White individuals which may account for their reduced odds of testing positive for SARS-CoV-2 in this analysis^17^. An additional hypothesis is that SARS-CoV-2 cases among Asian individuals might be underreported to VA as Asian groups may experience differential barriers to care and have a lower likelihood of utilizing VA services, including getting tested at VA, than White individuals^5,10,19^.

Our findings of racial and ethnic disparities for testing positive for SARS-CoV-2 provide important insight to help tailor strategies to contain and prevent further outbreaks in the United States. Early in the pandemic, tailored interventions to groups with higher risks may have been most effective. Now that the epidemic has generalized from large metropolitan centers with very high incidence to a more consistent rate of incidence across the country, racial and ethnic groups may be affected more equally suggesting that widescale prevention interventions for all persons may be most effective. However, the persistent disparities among Hispanic groups suggest that focused assessment and data-informed interventions in partnership with affected communities remains important in the effort to curtail infection hotspots, such as those due to a SARS-CoV-2 resurgence from the Delta and Omicron variants. SARS-CoV-2 is impacting all communities and is now much less concentrated in specific vulnerable groups compared to early in the pandemic. This does not imply that the overall cumulative burden of COVID-19 may be equal, as marginalized populations such as persons of color experienced substantial excess rates earlier in the epidemic and may experience excess extended effects or more severe outcomes from infection^20^. Of note, access to free or subsidized care at VA has reduced the impact of negative social determinants of health on SARS-CoV-2 outcomes as prior reports found no racial or ethnic disparities in mortality among patients who tested positive for SARS-CoV-2 in the VA^4^.

The VA electronic health record database offers the single largest nationwide data resource available in the United States with the required information on system-wide testing and detailed medical histories to examine racial and ethnic disparities. Our analysis identified time and regional variation in racial and ethnic disparities in SARS-CoV-2 positivity over the first 18 months of the pandemic independent of underlying health status and other key factors in a large, nationwide cohort. However, our analysis should be interpreted with some limitations, including those we previously described in detail^4,5^. In brief, we only examined tests administered in VA; therefore, our results may not be representative of all Veterans tested for SARS-CoV-2. Second, although this population was primarily male, it included over 140,000 women. Third, as is the case with most electronic health record data sources, we did not have the necessary information to account for all social determinants of health (e.g., occupation or household details) in our analysis, which are critical to understanding and preventing health inequities, particularly in infectious disease outbreaks. Fourth, SARS-CoV-2 test availability varied by local case load, facility supply, and local policy, particularly in the first wave when non-Hispanic Black and Hispanic patients were more likely to receive a SARS-CoV-2 test than non-Hispanic White patients^12^. However, after the first wave, testing was similar among all race and ethnic groups and test availability was unlikely to influence our results. Finally, while misclassified or missing data on race and ethnicity are often a concern, in this cohort, race and ethnicity were self-reported and there was a very small percentage with missing data. Focused research is needed to evaluate the association between social determinants of health and disparities seen during the COVID-19 pandemic as they may operate as confounders or may be on the causal pathway.

## Data Availability

Due to US Department of Veterans Affairs (VA) regulations and our ethics agreements, the analytic data sets used for this study are not permitted to leave the VA firewall without a Data Use Agreement. This limitation is consistent with other studies based on VA data. However, VA data are made freely available to researchers with an approved VA study protocol. For more information, please visit https://www.virec.research.va.gov or contact the VA Information Resource Center at VIReC@va.gov.
